# Evaluation of the Hologic Aptima® Vaginitis and Seegene Allplex™ assays for microbiological diagnosis of bacterial vaginosis and Candida vaginitis in women in France, relative to Nugent score and culture

**DOI:** 10.1099/jmm.0.002210

**Published:** 2026-10-20

**Authors:** Cécile Martaresche, Florian Scherrer

**Affiliations:** 1 Clinical Laboratory, INOVIE Barla, Nice, France

**Keywords:** Aptima bacterial vaginosis (BV), Aptima *Candida *vaginitis/*Trichomonas *vaginitis (CV/TV), bacterial vaginosis, diagnostic accuracy study, Nugent score, Seegene Allplex, sensitivity, specificity, vaginitis

## Abstract

**Introduction.** Molecular assays such as the Hologic Aptima^®^ Vaginitis Panel and Seegene Allplex^™^ Vaginitis assay offer improved sensitivity and multiplex detection for the diagnosis of vaginitis compared with the non-molecular standard of care.

**Gap Statement.** Evidence comparing the relative performance of Aptima versus Seegene is limited, as is knowledge of Aptima’s compatibility with non-proprietary swabs.

**Aim.** This prospective study assessed the diagnostic performance of the Aptima BV (bacterial vaginosis) and Aptima CV/TV assays (*Candida* vaginitis/*Trichomonas* vaginitis) alongside the Seegene Allplex assay in symptomatic women (≥18 years) presenting for vaginitis testing in primary care in France.

**Methodology.** Two vaginal swabs were taken per patient: one Aptima Multitest Swab and one non-proprietary Σ-TRANSWAB liquid Amies collection swab. Aptima swab data were used solely to confirm performance of the Aptima assays under current test conditions, while all primary assay comparisons were performed using Σ-TRANSWAB samples. These comparisons were performed for each assay relative to Nugent score (for BV) or culture (for CV), as well as Aptima relative to Seegene.

**Results.** In total, 500 women underwent vaginitis testing between July and August 2024. When using non-proprietary Σ-TRANSWAB collection methods, sensitivity and specificity values for the Aptima BV assay were 96.7 and 89.7% relative to Nugent score, and 89.9 and 90.6% for Seegene Allplex relative to Nugent score. Among ‘intermediate BV’ samples (*n*=66), agreement between Seegene Allplex and Nugent score was 1.5%. Sensitivity and specificity values for the detection of CV were 88.6 and 90.8% for the Aptima CV/TV assay, and 90.2 and 93.7% for Seegene Allplex. Assay detection of TV could not be validated due to an insufficient number of TV-positive samples (*n*=1). These findings were consistent with Aptima’s performance when using proprietary collection methods.

**Conclusion.** Overall, the Aptima Vaginitis Panel demonstrated comparably high diagnostic performance to Seegene Allplex for the detection of BV and CV when using a non-proprietary sample collection method. TV prevalence was too low in this cohort to permit meaningful assay evaluation for this pathogen.

**Impact Statement**

Findings from this study provide evidence to inform clinical decision-making for the diagnosis of bacterial vaginosis and *Candida* vaginitis in France. The comparable performance of the Aptima Vaginitis Panel and Seegene Allplex assays relative to standard-of-care methods, together with the compatibility of the Aptima assays with non-proprietary Σ-TRANSWAB^®^ swabs, supports consideration of molecular testing within flexible laboratory workflows and multi-assay testing from a single specimen. The very limited agreement between Seegene Allplex and Nugent scores for samples classified as ‘intermediate BV’ highlights diagnostic uncertainty surrounding this category and the need for greater standardization.

## Introduction

Vaginitis is a common gynaecological condition characterized by inflammation of the vagina [1]. Symptoms include abnormal discharge, odour, pruritus and burning [2]. Where a microbiological cause is identified, the most common causes of infectious vaginitis are bacterial vaginosis (BV; 40–50% of cases), *Candida* vaginitis (CV; 20–25%) and *Trichomonas* vaginitis (TV; 15–20%), all of which result in a shift in the composition of the vaginal microflora and disruption to vaginal health [3].

BV is defined by vaginal dysbiosis, whereby the normal *Lactobacillus*-dominated microbiota shifts towards an anaerobe-enriched environment, dominated by genera including *Gardnerella, Atopobium*, *Mobiluncus*, *Prevotella*, *Bacteroides* and *Peptostreptococcus* species [4–6]. In CV, >90% of infections are due to *C. albicans*, with the remainder consisting of *C. glabrata* and other species [7]. The anaerobic protozoan *T. vaginalis* is the cause of TV [2].

Although treatment of vaginitis can achieve high cure rates, recurrent and persistent infections are common [8–10]. Carriers can also be asymptomatic, often remaining undiagnosed and untreated until their condition is picked up during routine testing for sexually transmitted infections (STIs) [11]. Complications of undertreated vaginitis can be severe, such as pelvic inflammatory disease, which can be associated with chronic pelvic pain, ectopic pregnancy and infertility [12]. In pregnancy, BV and TV have been linked to complications such as premature birth, low birth weight and infections of the amniotic fluid [1, 13]. Chronic vaginitis also increases the risk of contracting and transmitting STIs [1, 13, 14]. As such, early and reliable diagnosis is critical to informing appropriate treatment and achieving optimal outcomes for patients.

In France, BV is routinely diagnosed using either Nugent or Hay–Ison scoring of Gram-stained vaginal smears [15, 16]. The Nugent system assigns a score from 0 to 10, reflecting the relative abundance of *Lactobacillus* versus BV-associated morphotypes [15]. Hay–Ison scoring classifies Gram-stained vaginal smears into four categories (Grades 0–III), reflecting the spectrum from normal *Lactobacillus*-predominant flora to BV-associated flora [16]. For CV, microscopy is used in first-line diagnostics, with culture reserved for species identification where required [17]. Wet mount microscopy or culture can still be used for the diagnosis of TV, though molecular diagnostic assays, such as nucleic acid amplification tests, are now recommended in European guidelines [17].

Each of the aforementioned non-molecular methods faces challenges in practice. Microscopy depends on technical expertise and has variable sensitivity, while Nugent scoring is inherently subjective and often requires consensus between reviewers [18–20]. CV culture is also resource-intensive and may fail to reliably identify non-albican*s* species [21, 22]. Lastly, TV detection by wet mount is highly time-sensitive, requiring immediate examination of fresh samples [22, 23]. As such, the latter method is often associated with reduced sensitivity due to organism degradation during transportation [22, 23].

In recent years, commercially available molecular diagnostic assays have been developed to address these limitations [19, 22, 24–31]. Compared with conventional diagnostics, molecular assays aim to offer enhanced specificity and sensitivity, with the potential to detect multiple pathogens simultaneously. The Hologic Aptima^®^ BV and Aptima^®^ CV/TV multiplex assays (Aptima^®^ Vaginitis Panel) use transcription-mediated amplification (TMA) of ribosomal RNA rRNA targets from vaginal swabs collected via Hologic’s proprietary Aptima^®^ Multitest Swab Specimen Collection Kit and processed on the Hologic Panther^®^ platform [32, 33]. In Canada, the USA, the UK and France, Aptima Vaginitis assays have demonstrated sensitivities of 91.1–98.8% and specificities of 77.7–96.3%, relative to standard-of-care (SOC) diagnostics [22, 34–37].

By contrast, the Seegene Allplex^™^ Vaginitis assay uses multiplex real-time (RT) polymerase chain reaction PCR to simultaneously detect the major pathogens that cause vaginitis [27, 38]. It uses Seegene’s automated All-In-One Platform, which includes automatic interpretation of infectious vaginitis. Vaginal swabs for testing with this assay can be collected using standard collection methods (i.e. non-proprietary kit).

Despite the availability and clinical use of both CE-approved assays in France, published evidence comparing the performance of both assays is currently limited to one relatively small study, which focused solely on BV, as opposed to the full spectrum of infectious vaginitis [39]. While the aforementioned study also included an analysis of non-proprietary swab use, further evidence is needed to better characterize Aptima’s performance with routinely used swabs and its potential impact on laboratory workflow.

Therefore, the present study compared the diagnostic performance of the Hologic Aptima BV and Aptima CV/TV assays and the Seegene Allplex Vaginitis assay against reference standards of Nugent scoring and culture, using the Σ-TRANSWAB^®^ collection method. These findings aim to inform evidence-based optimization of vaginitis diagnostics in France.

## Methods

### Study design

This was a prospective, diagnostic accuracy study conducted to evaluate the service and performance of the Aptima Vaginitis Panel (Hologic, Inc., San Diego, CA, USA) and the Seegene Allplex Vaginitis assay (Seegene, Inc., Seoul, South Korea), using residual samples taken for routine diagnostic testing. Assay performance was evaluated by direct comparison between assays, as well as comparison of each assay with Nugent scoring (BV) or culture methods (CV) as the reference diagnostic SOC.

### Sample collection

Samples were taken from women (assigned female at birth) aged ≥18 years with symptoms of vaginitis. Two vaginal swabs were taken per patient: one Aptima Multitest Swab (Hologic Inc., San Diego, CA, USA) and one non-proprietary Σ-TRANSWAB liquid Amies collection swab (Medical Wire and Equipment Co Ltd, Wiltshire, UK). Microscopy slides for Nugent scoring were also prepped from the same Σ-TRANSWAB collection tube. For the purpose of the key comparisons reported in this study, both assays were tested using the Σ-TRANSWAB collection method. Testing using the proprietary Aptima Multitest Swab was performed solely to provide contextual confirmation that the performance of the Aptima Vaginitis Panel was consistent with previously published data under the current study conditions; however, these data were not included as part of a formal comparative analysis between collection methods. For testing with the Aptima assays, 200 µl of the Σ-TRANSWAB solution was transferred into specimen transport media (Hologic, Inc., San Diego, CA, USA) prior to immediate testing on the appropriate detection system, representing an off-label adaptation of the manufacturer-recommended sample collection workflow. Samples were stored at room temperature and processed within 24 h of collection.

### Aptima Vaginitis Panel

The Aptima Vaginitis Panel encompasses two discrete assays, Aptima BV and Aptima CV/TV, which were performed according to the manufacturer’s protocol using the Hologic Panther^®^ system for detection (Hologic, Inc., San Diego, CA, USA). These assays use TMA of target rRNA to detect the relative abundance of *Lactobacillus* species in comparison to *Atopobium vaginae* and *Gardnerella vaginalis*. The multiplex Aptima CV/TV assay enables the detection of *Candida* species, with combined identification of *C. albicans*, *C. tropicalis*, *C. parapsilosis* and *C. dubliniensis*, while also enabling discrete detection of *C. glabrata* and *T. vaginalis*. Each assay includes an RNA-based internal control to verify successful amplification and absence of inhibition, as well as measuring human RNase P as an indicator of sample adequacy and cellularity.

### Seegene Allplex

The Seegene Allplex assay was performed in accordance with the manufacturer’s protocol (Seegene, Inc., Seoul, South Korea). Similarly to the Aptima BV assay, Seegene’s multiplex RT PCR assay measures relative levels of *Lactobacillus* species, *A. vaginae* and *G. vaginalis* to determine the presence of BV, though it can also detect *Mobiluncus* species. Detection of *C. albicans*, other *Candida* species (*C. krusei*, *C. glabrata*, *C. dubliniensis*, *C. parapsilosis*, *C. tropicalis* and *C. lusitaniae*) and *T. vaginalis* is used to identify CV and TV, respectively. Each Seegene Allplex assay incorporates an exogenous DNA-based internal control added to every reaction to monitor the efficiency of nucleic acid extraction, PCR amplification and inhibition.

### Diagnostic SOC

Nugent scoring of Gram-stained slides was performed by one scorer to determine the presence of BV. Scores range from 0 to 10 (0–3, normal flora; 4–6, intermediate or mixed flora; 7–10, BV-positive). In the case of any discrepancy between Nugent score and assay results, a second independent person was asked to review to determine the final result. For the diagnosis of CV, *Candida* species were identified by standard culture methods, using chromogenic medium for sample collection (bioMérieux, Lyon, France). All reviewers were blinded to the results of the molecular assays.

### Key objectives

The key objectives of this study were to report the sensitivity, specificity, positive predictive values (PPVs), negative predictive values (NPVs) and diagnostic accuracy of the Aptima Vaginitis Panel for the diagnosis of vaginitis when using an Σ-TRANSWAB for sample collection, relative to SOC or Seegene Allplex. We also describe similar performance metrics for the Seegene Allplex assay relative to SOC. In addition, an exploratory objective was to describe the agreement of the intermediate classification for BV between Nugent score and Seegene Allplex, in order to evaluate the consistency and interpretability of intermediate diagnostic categories across both approaches.

### Statistical analysis

Analyses of paired data were performed to calculate the sensitivity, specificity, PPVs and NPVs. The diagnostic accuracy of each test method was then calculated relative to a reference of either SOC method. The study design was not powered for inferential statistics; therefore, descriptive analyses were implemented as follows: cross-tabulations were used to examine the relationship between positive and negative results of each diagnostic method. Concordance results are presented with 95% CIs, which were estimated using the percentile bootstrap method with 2,000 resampling iterations.

## Results

### Study disposition

Genital swabs from 500 women were received for vaginitis testing during the study period (2 July 2024 to 24 August 2024), resulting in 500 Σ-TRANSWAB samples. Only paired samples were included for analysis (Aptima BV assay: *n*=431; Seegene Allplex assay: *n*=430). No further demographic data were collected.

### Sensitivity and specificity of Aptima BV and Seegene Allplex for the detection of BV

The clinical performance of the Aptima BV assay and the Seegene Allplex assay for the detection of BV when samples were collected by Σ-TRANSWAB is displayed in Table 1. Overall, both the Aptima BV and Seegene assays demonstrated high diagnostic value for the diagnosis of BV, with generally comparable performance between assays (Table 1). However, there was a small numerical increase in the sensitivity of the Aptima assay when referenced to Nugent score, compared with the sensitivity of Seegene referenced to Nugent (Table 1).

**Table 1.** Overall assay performance for the detection of BV

Assay comparison	N	TP	FN	TN	FP	Equivocals, n (%)	Sensitivity (%) (95% CI)*	Specificity (%) (95% CI)*	PPV (%) (95% CI)^†^	NPV (%) (95% CI)^†^
Aptima BV vs Seegene assay^‡^ (minimum performance)	431	120	14	276	21	9 (2.1)	89.6 (83.2, 93.7)	92.9 (89.4, 95.3)	85.1 (79.0, 90.7)	95.2 (92.6, 97.5)
Aptima BV vs Seegene assay^‡^ (maximum performance)	431	123	8	282	18		93.9 (88.4, 96.9)	94.0 (90.7, 96.2)	87.2 (81.5, 92.6)	97.2 (95.2, 99.0)
Aptima BV vs Seegene assay^‡^ excluding equivocals	422	120	8	276	18		93.8 (88.2, 96.8)	93.9 (90.5, 96.1)	87.0 (81.1, 92.4)	97.2 (95.1, 99.0)
Aptima BV vs Nugent score (minimum performance)	431	88	37	253	53	58 (13.5)	70.4 (61.9, 77.7)	82.7 (78.0, 86.5)	62.4 (54.5, 70.6)	87.2 (83.2, 91.0)
Aptima BV vs Nugent score (maximum performance)	431	112	3	287	29		97.4 (92.6, 99.1)	90.8 (87.1, 93.5)	79.4 (72.4, 85.9)	99.0 (97.6, 100)
Aptima BV vs Nugent score excluding equivocals	373	88	3	253	29		96.7 (90.8, 98.9)	89.7 (85.6, 92.7)	75.2 (66.9, 83.0)	98.8 (97.3, 100)
Seegene assay vs Nugent score (minimum performance)	430	80	46	250	54	57 (13.3)	63.5 (54.8, 71.4)	82.2 (77.5, 86.1)	59.7 (51.1, 67.7)	84.5 (80.0, 88.3)
Seegene assay vs Nugent score (maximum performance)	430	104	9	291	26		92.0 (85.6, 95.8)	91.8 (88.3, 94.3)	80.0 (73.0, 87.0)	97.0 (94.8, 98.7)
Seegene assay vs Nugent score excluding equivocals	365	80	9	250	26		89.9 (81.9, 94.6)	90.6 (86.6, 93.5)	75.5 (67.3, 83.7)	96.5 (93.9, 98.5)

*Score CIs.

†CIs obtained using the percentiles of the bootstrap resampling distribution with 2,000 iterations.

‡Since Seegene Allplex is not regarded as the ‘gold standard’ for diagnosing vaginitis, the terms PPA and NPA were used in place of sensitivity and specificity, respectively, to describe the corresponding data for this comparison. All samples were collected using the Σ-TRANSWAB collection method. The Seegene assay and Nugent score results both include an intermediate zone. When intermediate results are included in the analysis, they are treated as equivocal results. Analyses using minimum performance of equivocals treat all equivocals as discordant results (i.e. FN and FP). Analysis using maximum performance of equivocals treats all equivocals as concordant results (i.e. TN and TP).

BV, bacterial vaginosis; CI, confidence interval; FN, false negative; FP, false positive; N, number of samples; NPA, negative percent agreement; PPA, positive percent agreement; TN, true negative; TP, true positive.

Briefly, relative to the Nugent score and excluding equivocals, the sensitivity values were 96.7% for Aptima and 89.9% for Seegene, while specificities were 89.7 and 90.6% for Aptima and Seegene, respectively. PPVs were 75.2% for Aptima BV and 75.5% for Seegene, while NPVs were 98.8 and 96.5%, respectively (Table 1).

When comparing the Aptima BV assay to Seegene Allplex, data are reported as positive percent agreement (PPA) and negative percent agreement (NPA), rather than sensitivity and specificity, to reflect that neither assay is considered a definitive reference standard. Excluding equivocals (*n*=422), the Aptima BV assay showed a PPA of 93.8% and an NPA of 93.9%, relative to Seegene (Table 1). PPVs and NPVs of this comparison were 87.0 and 97.2%, respectively, indicating good agreement between both molecular assays (Table 1).

When using the proprietary Aptima Multitest Swab Collection Kit with the Aptima BV assay, the diagnostic performance was as previously reported, with a sensitivity value of 89.0% and specificity of 93.3%, relative to Nugent score (data not shown).

### Comparison of intermediate classifications between Nugent score and Seegene Allplex

Overall, 66 samples from our study were classified as intermediate BV by either Nugent score or Seegene Allplex (Table S1, available in the online Supplementary Material). Among those classified as intermediate by Nugent score (*n*=58), only one sample was also classified as intermediate by Seegene, while the majority were classified as negative (*n*=35) or positive (*n*=22) by the latter assay. Similarly, among the Seegene intermediate samples (*n*=9), six were negative and two were positive by Nugent score. Across all 66 samples, the level of agreement between the two methods for intermediate BV categorization was 1.5%, with disagreement observed in 98.5% of samples (Table S1).

### Sensitivity and specificity of Aptima CV/TV and Seegene Allplex for the diagnosis of CV

The clinical performance of the Aptima CV/TV and Seegene Allplex assays for the detection of CV using Σ-TRANSWAB-collected samples is displayed in Table 2. Overall, both the Aptima CV/TV (*n*=428) and Seegene (*n*=428) assays demonstrated high sensitivity and specificity for the detection of CV, compared with culture methods, though the performance of the Seegene assay was slightly numerically higher than that of Aptima CV/TV (sensitivity: Aptima, 88.6%; Seegene Allplex, 90.2%; specificity: Aptima, 90.8%; Seegene Allplex: 93.7%; Table 2). For comparison of the Aptima CV/TV assay relative to Seegene (*n*=428), the PPA was 88.4% and NPA was 93.2% (Table 2).

**Table 2.** Assay performance for the detection of CV

Assay comparison	N	TP	FN	TN	FP	Sensitivity (%) (95% CI)*	Specificity (%) (95% CI)*	PPV (%) (95% CI)†	NPV (%) (95% CI)†
Aptima CV/TV vs culture	428	101	13	285	29	88.6 (81.5, 93.2)	90.8 (87.1, 93.0)	77.7 (70.5, 84.8)	95.6 (93.2, 97.7)
Seegene assay vs culture	428	101	11	296	20	90.2 (83.3, 94.4)	93.7 (90.4, 96.0)	83.5 (76.7, 89.8)	96.4 (94.2, 98.4)
Aptima CV/TV vs Seegene‡	428	107	14	286	21	88.4 (81.5, 93.0)	93.2 (89.8, 95.0)	83.6 (77.2, 90.0)	95.3 (92.9, 97.7)

*Score CIs

†CIs obtained using the percentiles of the bootstrap resampling distribution with 2,000 iterations.

‡Since Seegene Allplex is not regarded as the ‘gold standard’ for diagnosing vaginitis, the terms PPA and NPA were used in place of sensitivity and specificity, respectively, to describe the corresponding data for this comparison. All samples were collected using the Σ‑TRANSWAB collection method. The Seegene assay and Nugent score results both include an intermediate zone. When intermediate results are included in the analysis, they are treated as equivocal results. Analyses using minimum performance of equivocals treat all equivocals as discordant results (i.e. FN and FP). Analysis using maximum performance of equivocals treats all equivocals as concordant results (i.e. TN and TP).

CI, confidence interval; CV, *Candida *vaginitis; FN, false negative; FP, false positive; N, number of samples; NPA, negative percent agreement; NPV, negative predictive value; PPA, positive percent agreement; PPV, positive predictive value; TN, true negative; TP, true positive; TV, *Trichomonas *vaginitis.

When using the proprietary Aptima Multitest collection method with the Aptima CV/TV assay, the diagnostic performance for the detection of CV was as previously reported, with sensitivity and specificity values of 89.5 and 87.3%, respectively, relative to culture (data not shown).

The clinical performance of both assays for the detection of TV was also explored; however, due to the low prevalence of TV within our sample (*n*=1), performance metrics for TV could not be reliably estimated.

### Detection of *Mobiluncus* species by Seegene Allplex

Unlike the Aptima Vaginitis Panel, the Seegene Allplex assay also detects *Mobiluncus* species as an additional marker of BV. Testing of samples for BV via Seegene or Nugent scoring revealed a substantially higher overall incidence of *Mobiluncus-*negative (*Mob*^-ve^) versus *Mobiluncus*-positive (*Mob*^+ve^) samples in our cohort, irrespective of test method (Seegene: *Mob*^-ve^ *n*=360, *Mob*^+ve^ *n*=70; Nugent: *Mob*^-ve^ *n*=410, *Mob*^+ve^ *n*=22; Fig. 1a). Furthermore, while *Mobiluncus* was detected in some samples classed as BV-positive by either Seegene or Nugent score (score of 7–10; Seegene: *n*=36; Nugent: *n*=20), a higher frequency of BV-positive samples were in fact *Mob^-^*^ve^ (Seegene: *n*=92; Nugent: *n*=71; Fig. 1a). Similarly, *Mobiluncus* was detected in 36 samples classed as BV-positive by both Aptima and Seegene (score of 7–10); however, 84 samples that were BV-positive under the same assay conditions were *Mob^-^*^ve^ (Fig. 1b).

**Fig. 1.**
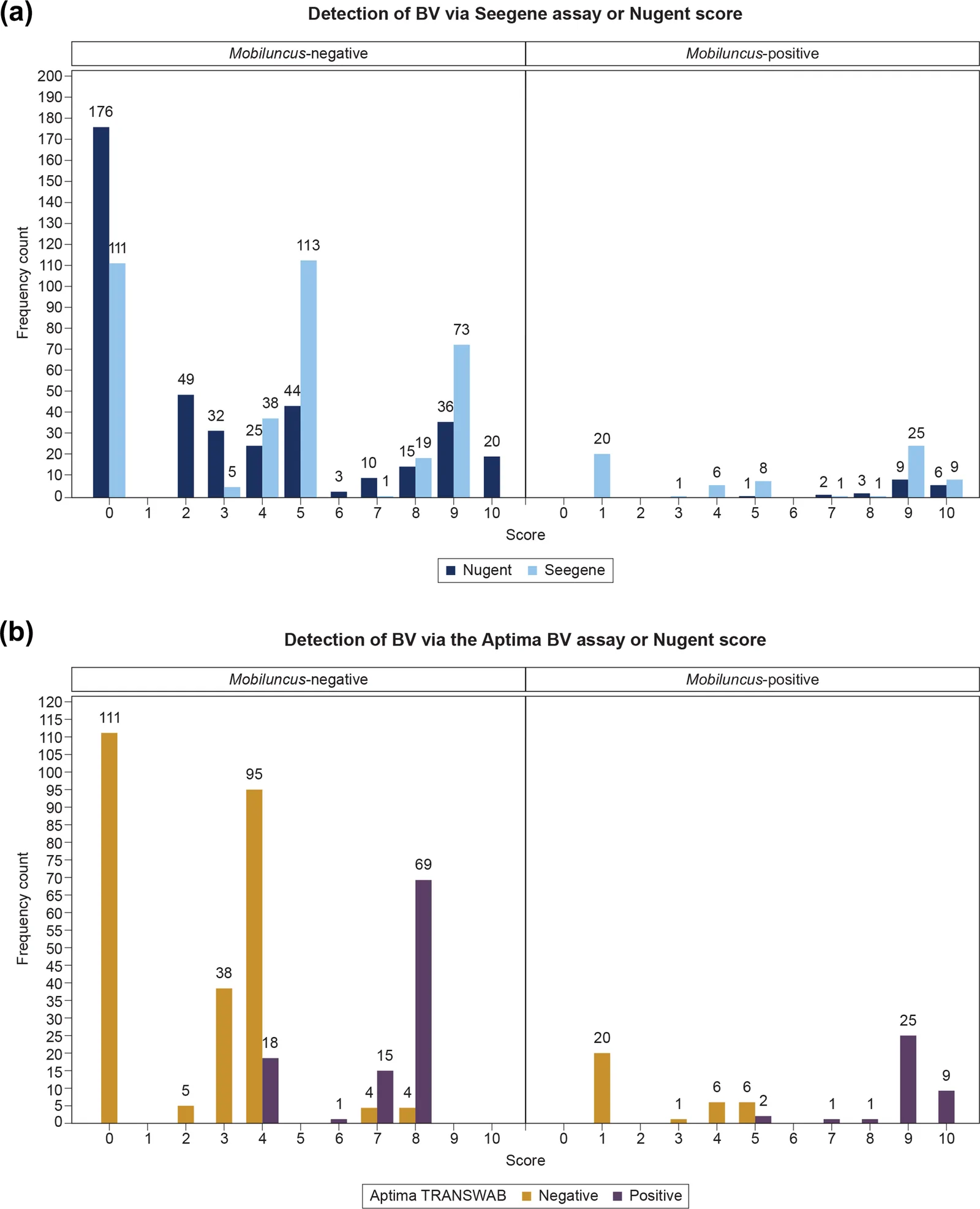
Detection of *Mobiluncus* species in samples tested for bacterial vaginitis. The presence or absence of *Mobiluncus* species was assessed in samples tested for BV using the Seegene Allplex assay or Nugent score (**a**), and in samples tested for BV using the Aptima BV RT-TMA assay (quantitatively detects *Lactobacillus* species, *G. vaginalis* and *A vaginae*) or Nugent score (**b**). A positive diagnosis of BV was determined using the Nugent score (0–3, normal; 4–6, intermediate; 7–10, BV) or the Seegene assay (0–4, normal; 5–6, intermediate; 7–10, BV). Notably, the Aptima BV assay cannot detect the presence of *Mobiluncus* species.

Regarding samples classed as intermediate BV using Seegene (score of 5–6) or Nugent score (score of 4–6), among *Mob*^-ve^ samples, 57 were classed as intermediate using Nugent score, whereas only 1 sample fell into this category using Seegene Allplex (Fig. 1a). For *Mob*^+ve^ samples, only one sample was classified as intermediate BV by Nugent scoring, while eight samples were intermediate via the Seegene assay. As per findings for the overall cohort, these data indicate poor agreement between both assays regarding intermediate BV categories.

## Discussion

We set out to evaluate the clinical performance of the Aptima Vaginitis Panel and the Seegene Allplex assay for the diagnosis of infectious vaginitis in women attending primary care centres in France. Previous studies have demonstrated the robust performance of the Aptima Vaginitis Panel using its proprietary Aptima Multitest swab, with high sensitivities and specificities for the detection of BV, CV and TV [22, 35, 36]. In the current study, our results demonstrate the high clinical performance of both assays for the detection of BV and CV, even when using a non-proprietary sample collection method (Σ-TRANSWAB) with the Aptima assays.

In particular, regarding the detection of BV, both assays reported sensitivities and specificities of ~90% or above, relative to Nugent score. Sensitivity for the Aptima BV assay relative to Nugent score was numerically higher than that of Seegene relative to Nugent, at 97% versus 90%. However, it is important to note that this study was descriptive in nature; therefore, further appropriately powered statistical analyses would be required to confirm potential performative differences between both assays. When the evaluation of Aptima BV was performed relative to the Seegene Allplex assay, the Aptima BV assay demonstrated a consistently high performance, with PPA and NPA values ≥90%.

When testing for CV, we observed similarly robust and comparable performances for both assays relative to SOC culture, with sensitivities of 88.6% for the Aptima CV/TV assay and 90.2% for Seegene Allplex, and specificities of 90.8% for the Aptima CV/TV assay and 93.7% for Seegene Allplex. With respect to TV, we observed an extremely low pervasiveness of TV among the sampled population (*n*=1), limiting any further evaluation of assay performance for the detection of TV. Notably, the low prevalence of TV in our study is in agreement with previous findings of a French cohort screened for STIs (*N*=41,507; TV-positivity rate of 0.1%) [40].

Although it was not the key focus of our analysis, we also performed evaluations of the Aptima Vaginitis Panel using its own proprietary Aptima Multitest Sample Collection Kit to provide context for our Σ-TRANSWAB findings. We report that the performance observed using the Aptima Multitest swab was consistent with that obtained using Σ-TRANSWAB samples, demonstrating high diagnostic accuracy irrespective of sample collection method.

Sensitivity and specificity data from the current study also align with previous reports from the literature. For example, the Aptima Vaginitis Panel has reported sensitivities ranging from 91.1 to 98.8% and specificities ranging from 77.7 to 96.3% relative to SOC using its own proprietary sample collection kit [22, 34–37]. Additionally, the Seegene Allplex assay has previously been reported to exhibit a sensitivity of 91.7% and specificity of 86.6% for the detection of BV, and 91.1 and 95.6% for the detection of CV [27]. Regarding the direct comparison of the Aptima Vaginitis Panel versus Seegene Allplex when using non-proprietary swabs for sample collection, a recent small-to-moderately sized study in the Netherlands reported a PPA of 77% and an NPA of 100% for the detection of BV [39]. The NPA observed in our study (97%) closely aligns with this finding, though the PPA was notably higher in our cohort (87% vs 77%). This difference may reflect variation in sample size, population characteristics or extraction protocols between studies, rather than a direct discrepancy in assay performance, and should be interpreted with caution given differences in study design. Collectively, these findings support the reproducibility of NPA and the overall comparability of both assays for the detection of infectious vaginitis across different study settings and sample collection approaches, despite some variability in PPA between cohorts.

However, even among distinct molecular assays with comparably high diagnostic accuracy, such as the Aptima Vaginitis Panel and Seegene Allplex assay, there are practical considerations that may influence the selection of one assay over another for a given laboratory. For example, use of a non-proprietary system, such as the Σ-TRANSWAB, allows a single sample to be deployed across multiple distinct assays, rather than requiring dedicated swabs for each test. This approach may help to reduce the need for repeat sampling and could improve overall efficiency in diagnostic settings. While the Seegene Allplex has previously been validated using a non-proprietary sample collection method [27], our study provides further evidence that the Aptima Vaginitis Panel performs consistently with both proprietary and non-proprietary sample collection methods for the detection of vaginitis [37]. These findings support the compatibility of Aptima with flexible sampling workflows already achievable with multiplex molecular platforms, such as Seegene Allplex, while also suggesting that laboratories may be able to select their preferred swab system when using the Aptima Vaginitis Panel without compromising diagnostic breadth across the spectrum of vaginal health testing. Notably, use of non-proprietary swabs with the Aptima Vaginitis Panel requires an additional step, whereby samples must be transferred into specimen transfer media prior to processing on the Panther detection system, which may introduce extra handling time in high-throughput settings.

Another key distinction between the Aptima Vaginitis Panel and the Seegene Allplex assay that may influence assay selection is the type of data output. The Aptima Vaginitis Panel provides a binary output (‘positive’ or ‘negative’), which may reduce interpretive complexity. In contrast, the Seegene Allplex assay provides detailed quantitative outputs, including *Ct* values and PCR curves, which may capture subtle shifts in vaginal microbiota, though these outputs require additional training and staff input to guide interpretation. While Seegene’s more nuanced output may be informative in research settings, in routine high-throughput diagnostic practice, the added complexity may not translate to improved patient management, potentially making binary output a more practical option for routine workflows in some laboratory settings. However, this simplicity may come at the cost of the additional biological detail captured by quantitative outputs, such as Seegene’s, which may be valuable in specific clinical or research contexts.

Aptima’s binary approach also does not include the intermediate category, unlike conventional Nugent scoring and the Seegene Allplex assay, each of which defines its own intermediate classification. While intermediate classifications may help to identify transitional or borderline states of vaginal dysbiosis, the clinical interpretation of these categories remains uncertain, which may contribute to diagnostic uncertainty [39]. In our study, we observed very limited overlap between samples classed as intermediate by Seegene versus Nugent score (1.5%), in line with previously published findings [27]. Though this likely reflects methodological differences between morphologic and molecular approaches to BV classification, it also highlights the lack of consistency in how intermediate categories are defined across diagnostic systems. Therefore, our observations suggest that the intermediate category may be highly assay-dependent, potentially contributing to limited interpretive consistency in routine clinical practice and the need for additional diagnostic review within laboratories.

Species identification is another factor to consider when selecting the most appropriate assay. As discussed throughout, the Seegene Allplex assay is capable of detecting the presence of *Mobiluncus* species, which have previously been reported to be associated with BV [41–43], whereas this marker is not included in the Aptima BV assay. As such, the broader microbiome profiling offered by Seegene could potentially detect atypical cases of BV that Aptima may miss, as well as guide antibiotic choice in recurrent/resistant cases. However, we detected a relatively low frequency of *Mobiluncus* positivity in our study. Moreover, BV-positive samples by Seegene, Aptima BV or Nugent score were more commonly *Mob*^-ve^ than *Mob*^+ve^. It is also worth noting that in our study, the capability to detect *Mobiluncus* did not confer any additional benefits relating to a gain in sensitivity for the detection of BV for Seegene versus Aptima. Collectively, our data suggest that this marker is not necessary for the detection of BV, though further large-scale studies are warranted to verify our findings.

As with all studies, ours was subject to some limitations. Most pertinent to our overarching comparison is that this study was not powered for the detection of statistical differences between paired data groups, meaning that any observed numerical differences in diagnostic accuracy should be considered exploratory in nature and interpreted with appropriate caution. Furthermore, no discrepancy analysis was performed on discordant results, though both molecular assays demonstrated strong overall agreement with SOC methods, supporting their clinical utility. A particular strength of our study is that, to the best of our knowledge, it provides one of the first direct comparisons of the overall clinical performance of the Aptima Vaginitis Panel with the Seegene Allplex assay for the detection of both BV and CV. We also demonstrate these findings in a relatively large cohort.

## Conclusions

Both the Aptima Vaginitis Panel and the Seegene Allplex assay demonstrate high diagnostic performance for the detection of BV and CV. Relative to SOC, performance was generally comparable between assays. Notably, the rarity of TV cases encountered during the study period meant that neither assay’s performance for this pathogen could be meaningfully assessed here, and dedicated studies in populations with higher TV burden remain needed. The Aptima Vaginitis Panel maintained its robust performance when using a non-proprietary Σ-TRANSWAB for sample collection, as opposed to Aptima’s proprietary sample collection kit, highlighting its clinical versatility and applicability across diverse laboratory workflows. Beyond diagnostic accuracy, practical factors such as sample collection flexibility, data output format and handling of intermediate or borderline results are likely to influence assay selection depending on laboratory context and workflow priorities. Larger, multi-site studies would help to confirm the generalizability of our findings and further clarify how these practical considerations translate into clinical and operational outcomes.

## Supplementary material

10.1099/jmm.0.002210 Table S1.

## Abbreviations

BV: bacterial vaginosis
CE: European conformity
CI: confidence interval
CV: *Candida* vaginitis
DNA: deoxyribonucleic acid
FN: false negative
FP: false positive
*Mob* ^-ve^: *Mobiluncus*-negative
*Mob* ^+ve^: *Mobiluncus*-positive
NPA: negative percent agreement
NPVs: negative predictive values
PCR: polymerase chain reaction
PPA: positive percent agreement
PPVs: positive predictive values
RNA: ribonucleic acid
rRNA: ribosomal ribonucleic acid
RT: real-time
SOC: standard-of-care
STIs: sexually transmitted infections
TMA: transcription-mediated amplification
TN: true negative
TP: true positive
TV: *Trichomonas* vaginitis
