# Supplementary material for: Evaluation of the Hologic Aptima® Vaginitis and Seegene Allplex™ assays for microbiological diagnosis of bacterial vaginosis and Candida vaginitis in women in France, relative to Nugent score and culture
Source: J Med Microbiol. 2026 Oct 20;75(10):002210. doi: 10.1099/jmm.0.002210 (PMC13682698; doi:10.1099/jmm.0.002210)
Supplement: Supplementary file 1 [file jmm-75-02210-s001.pdf]

SUPPLEMENT

Evaluation of the Hologic Aptima® Vaginitis and Seegene Allplex™ Assays for Microbiological Diagnosis of Vaginitis in Women in France, Relative to Nugent Score and Culture

Short running head: Vaginitis assay evaluation in France

Cécile Martaresche[1] and Florian Scherrer[1]

[1]Clinical Laboratory, INOVIE Barla, Nice, France

Supplemental Table S1. Comparison of intermediate BV category between Nugent score versus Seegene Allplex assay

	Seegene Allplex assay		
**Nugent score**	**Negative**	**Intermediate**	**Positive**
**Negative**	0	6	0
**Intermediate**	35	1	22
**Positive**	0	2	0

Samples were classed as ‘intermediate BV’ if they received a score of 4–6 using the Nugent score or a score of 5–6 when using the Seegene Allplex assay.
BV, bacterial vaginosis.
